# Bath, Contrexéville and the Lime Sulphated Waters

**Published:** 1886-06

**Authors:** 


*Bath, Contrexéville and the Lime Sulphated Waters.* By JOHN MACPHERSON, M.D. Pp. 52. London: H. K. LEWIS. 1886.

This is a useful and pleasantly written little work on a class of waters which are of the greatest therapeutic value. Analysis of the various springs, British and Continental, with descriptions of the various diseases which derive most benefit at the individual spas, and accounts of the climatic and local conditions which exist in their neighbourhood, form a guide book which will be highly appreciated by medical men.

The work has a local as well as a general interest. Hear what he says of our neglected Hotwells: "The idea has been frequently entertained of late, of endeavouring to restore the popularity of the old wells, but nothing has as yet been practically effected. To carry out such a plan, the two most necessary measures seem to be, to have the wells entirely protected from the tide, and to have their waters pumped up with as little loss of heat as possible to the top of the bank, for patients will always prefer to drink the water without having to ascend 200 feet or more, after their potations. Is there any probability of success in an attempt to restore the waters? It is hard to say. But the remarkable healthiness of Clifton is in its favour, and a great deal will depend on the management of the waters, on the arrangements made for the local treatment of the fauces and larynx, on assiduous advertising, and not a little on fashion. There is no reason, particularly when we look back to the past, why the scheme may not succeed, when we recollect that Evian, which is cold, and has a feebler mineralisation than the Hotwells, is considered to be a good substitute, in vesical affections, for Vichy and Contrexéville, and that lime-waters, like Lippspringe and Weissenburg, one a little warmer and one a little colder than the Hotwells, neither to my mind very attractive, enjoy a reputation in pulmonary complaints."

That the revival of the spa would be attended with success we have no doubt whatever. Baths are not, in the first inception at least, necessary. A pump-room, a good band, a small concert-room, and advertising, are all the essentials. The present Observatory, with very slight change, would form an excellent pump-room and concert-room. The water could easily be raised that height with little loss of heat. The situation of the Observatory is an ideal one for a pump-room. It is at a little distance from places of residence, and the useful morning walk is thereby rendered essential. It is in a breezy open situation, with a lovely view, where the rest after drinking is both beneficial and pleasant. It is surrounded by an open space forming a magnificent promenade, on which the morning stroll may be taken while the band plays. We venture to affirm that, as a site for a pump-room, the Observatory hill at Clifton is unrivalled in England. The Hotwells, with their two or three thousand gallons per hour of highly valuable medicinal waters, could easily be carried thither by a small engine through a metal tube an inch in diameter. If on the whole scheme our Corporation were to expend one half of the sum which our spirited neighbours at Bath expended the other day on a slight extension of theirs, we should have Clifton again a fashionable health resort.

The Hotwells water is peculiarly suited to the diseases of modern life; the climate and environs of Clifton are such as the hurried and jaded brain-worker of the nineteenth century longs for. It only wants (to put it from the taxpayers' point of view) the expenditure of half a dredger by the Corporation of Bristol to restore the past repute of the spa, and improve the future prospects of Clifton.

